# Research attitudes through the COVID-19 pandemic

**DOI:** 10.3389/fpubh.2026.1899763

**Published:** 2026-10-15

**Authors:** Sarah K. Schlund, Dan K. Hoang, Amy J. H. Kind, Joshua D. Grill, Daniel L. Gillen

**Affiliations:** 1 Department of Statistics, University of California, Irvine, Irvine, CA, United States; 2 Irvine Institute for Memory Impairments and Neurological Disorders, University of California, Irvine, Irvine, CA, United States; 3 Center for Health Disparities Research, University of Wisconsin School of Medicine and Public Health, Madison, WI, United States; 4 Division of Geriatrics, Department of Medicine, University of Wisconsin School of Medicine and Public Health, Madison, WI, United States; 5 Department of Neurobiology and Behavior, University of California, Irvine, Irvine, CA, United States; 6 Department of Psychiatry and Human Behavior, University of California, Irvine, Irvine, CA, United States; 7 Institute for Clinical and Translational Science, University of California, Irvine, Irvine, CA, United States

**Keywords:** attitudes towards research, clinical trials, COVID-19, recruitment, registries, research attitudes questionnaire

## Abstract

**Introduction:**

Identifying the factors that influence attitudes toward biomedical research is essential for improving public engagement and participation. The COVID-19 pandemic offers a unique opportunity to assess how a public health crisis shapes perceptions of clinical research. We investigated whether attitudes toward biomedical research differed across phases of the COVID-19 pandemic.

**Methods:**

We analyzed data from 5,098 adults enrolled in the UC Irvine Consent-to-Contact (C2C) registry, an online voluntary recruitment registry in Southern California, between June 2016 and August 2025. Research attitudes were assessed with the validated 7-item Research Attitudes Questionnaire (RAQ) at enrollment and annually thereafter. Scores were analyzed continuously (7 -35) and dichotomously, with >28 defined a priori as high. COVID-19 periods were defined a priori as: P1 (before March 13, 2020—the U.S. national emergency declaration), P2 (March 13 to December 11, 2020—prior to COVID-19 vaccine approval), and P3 (after December 11, 2020—post-vaccine approval). Cross-sectional analyses included all participants; longitudinal analyses were limited to 1,388 participants who enrolled before March 2020 and had ≥3 assessments.

**Results:**

Our sample of 5,098 adults was predominantly non-Hispanic White (70.2%), female (65.7%), highly educated, and over age 50 (66.7%). Participants enrolling during P2 had 60% higher odds of a high RAQ score (>28) compared with P1 (aOR 1.60; 95% CI: 1.07, 2.40; *p*=0.022). Longitudinally, the mean within-subject RAQ score was significantly higher during P2 versus P1 (Est. 0.37; 95% CI: 0.08, 0.66; *p*=0.011), and the odds of a high RAQ score were 24% higher in P2 (aOR 1.24; 95% CI: 1.09, 1.39; *p*<0.001) compared to P1.

**Discussion:**

These findings suggest that awareness of urgent medical issues, such as the COVID-19 pandemic, may affect attitudes toward research.

## Introduction

Adequately powered, well-controlled clinical trials are essential for advancing biomedical science. Trials provide the evidence needed to expand medical knowledge, develop new treatments, and improve patient care. Efficiently recruiting and retaining a representative group of participants is a consistent and major challenge in conducting trials (1). To develop more effective recruitment strategies, it is essential to first understand the factors that influence an individual’s willingness to participate in clinical research, including their attitudes toward research (2–6).

The COVID-19 pandemic presents a unique opportunity to understand how national news and public dialogue, in this case related to a public health crisis, may influence attitudes toward biomedical research. While the pandemic brought unprecedented attention to biomedical research (7), particularly vaccine development, it also fueled the spread of misinformation, raising public skepticism about research safety and efficacy (8). The simultaneous rise in public awareness of biomedical research and the spread of false information provide a compelling context to examine how public health emergencies influence perceptions of research and, ultimately, participation in clinical trials.

The Research Attitudes Questionnaire (RAQ) is a validated instrument designed to assess an individual’s views on biomedical research, with higher scores indicating more favorable views (9). The RAQ has been shown to be predictive of anticipated (2) and actual (3) clinical research enrollment, study compliance, and trial completion (10).

This study examined changes in research attitudes throughout the COVID-19 pandemic using RAQ scores collected from the UC Irvine Consent-to-Contact (C2C) Registry (11), a voluntary recruitment registry that connects potential participants with clinical research studies at UC Irvine. We tested whether RAQ scores differed across three periods—pre-pandemic (P1), early pandemic (P2), and post–vaccine availability (P3)—and hypothesized *a priori* that scores would be higher in P2 and P3 compared with P1.

## Materials and methods

### Data and sample

We analyzed data from 5,098 adults (aged 18 years or older) in Orange County, California, who voluntarily enrolled in the UC Irvine Consent-to-Contact (C2C) registry between June 1, 2016, and August 15, 2025. Upon enrollment, participants completed a survey capturing demographic and clinical information (11), as well as some structured elements, including the Research Attitudes Questionnaire (RAQ). They were asked to update their information annually. The RAQ was administered online across all study periods.

The C2C is approved by the UCI Institutional Review Board. All registrants provided informed consent upon enrollment in the C2C. Consent included permission to be referred to studies as well as to use enrollment data to answer novel research questions. The study findings are reported in accordance with Strengthening the Reporting of Observational Studies in Epidemiology (STROBE) reporting guideline (12). The current study was conducted in accordance with the ethical standards set forth in the WMA Declaration of Helsinki for research involving human participants.

For longitudinal analyses, we excluded individuals who enrolled after March 13, 2020, and those who had fewer than three RAQ assessments, resulting in a subsample of 1,388 participants. Among these participants, the average amount of follow-up was 4.18 years. All participants were included in analyses of initial RAQ scores.

Our primary outcome was the total score on the 7-item RAQ, a self-reported survey in which participants rate seven questions using a 5-point Likert scale from strongly disagree (1) to strongly agree (5) (9). RAQ scores therefore range from 7 to 35, with higher scores reflecting more favorable attitudes toward biomedical research.

Our secondary outcome was a dichotomized total RAQ score, coded as 0 for scores ≤28 and 1 for scores >28. We selected the 28-point cut point because, on the 5-point response scale, a total >28 corresponds to an average item score >4, reflecting predominantly favorable (agree/strongly agree) responses. This dichotomization aligns with prior studies (10, 13–15) and is supported by the observed distribution of baseline RAQ scores in our sample (Figure 1).

**Figure 1:**
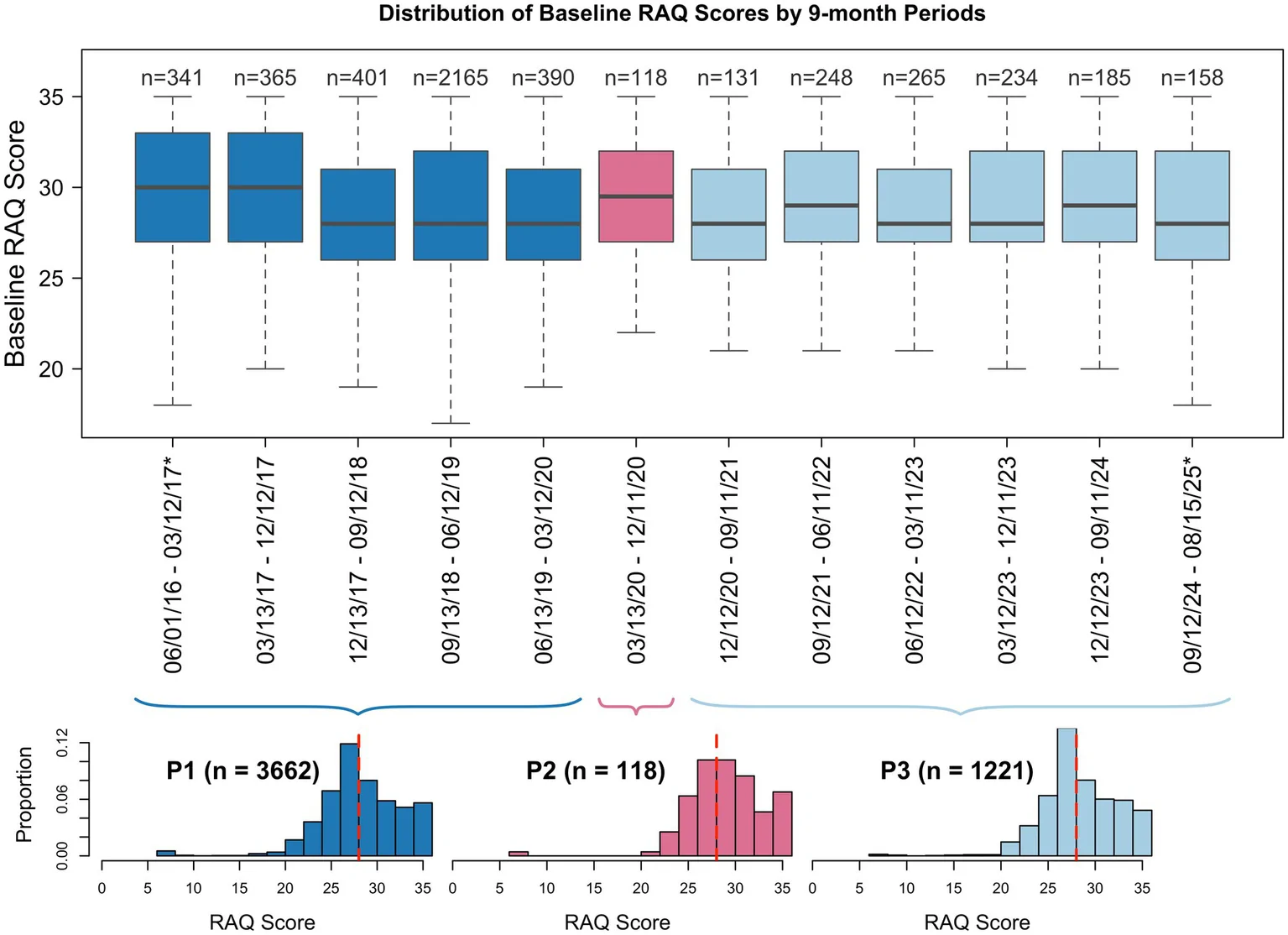
Temporal trend and distributions of RAQ scores at enrollment across study periods. The top panel displays boxplots of initial RAQ scores in 9-month intervals, centered around the early pandemic period (P2; March 13–December 11, 2020, highlighted in pink). The bottom panel displays histograms of RAQ scores at enrollment for each COVID-19 time period: pre-pandemic (P1), early pandemic (P2), and post-vaccine availability (P3). Dashed red lines indicate a RAQ score of 28, the threshold for a “high” score. *The first and last box-plot intervals were extended beyond 9 months to include all available observations.

Our predictor of interest was a categorical variable with three levels representing distinct phases of the COVID-19 pandemic, defined *a priori* as: P1, before March 13, 2020, the date the U.S. declared COVID-19 a National Emergency (16); P2, March 13 to December 11, 2020, the date the FDA issued the first emergency use authorization for a COVID-19 vaccine (17); and P3, after December 11, 2020.

Adjustment covariates included age, recruitment source, sex, race and ethnicity, years of education, presence of a chronic health condition, and Area Deprivation Index (ADI) (18, 19). Age was collected as a continuous variable, with participants over 90 categorized as “90+” (*n* = 42) to protect their identities and coded as 91 for the purpose of analysis. Recruitment source captured how participants first learned about C2C, including email, physician referral, friend referral, community talk, newspaper, postcard, TV, radio, online search, social media, and none of the above. For analysis, email and physician referral were retained as separate categories; the remaining options were grouped into personal network (friend referral, community talk), media/online (newspaper, TV, radio, online search, social media), and other (postcard, multiple sources, none of the above). We used chronic health condition as a binary variable based on self-reported diagnoses of cancer, liver or kidney disease, heart failure, coronary artery disease, diabetes, hypertension, or hypercholesterolemia. Education was recorded continuously; we recoded values greater than 26 (*n* = 33) to 27. ADI, which measures neighborhood disadvantage, was based on California state rankings and incorporated as quintiles, with 1 indicating the least disadvantaged and 5 the most (18). Race and ethnicity were grouped based on self-report as non-Hispanic (NH) White, Hispanic White, NH Asian, NH Black, and Other (including Hispanic Black (*n* = 2), Hispanic Asian (*n* = 5), NH/Hispanic American Indian or Alaska Native (*n* = 17), NH/Hispanic Native Hawaiian or Other Pacific Islander (*n* = 9), self-identified other (*n* = 209), and multiracial (*n* = 198)). Participants identifying sex as “Other” (*n* = 11) or declining to disclose race/ethnicity (*n* = 166) were treated as missing for those variables.

### Statistical analysis

The participant sample was characterized using summary statistics, stratified by pandemic phase. For continuous baseline covariates, means and standard deviations are presented, while for discrete baseline covariates, frequencies and percentages are reported.

We assessed changes in RAQ scores using both cross-sectional and longitudinal approaches. First, we assessed the association between RAQ score at time of enrollment and pandemic period. We fit a linear regression model with P1 as the reference group, using Huber-White robust standard errors to guard against heteroskedasticity (20). We adjusted for the following *a priori* identified potential confounding factors: age, sex, years of education, recruitment source, race and ethnicity, presence of a chronic health condition, and ADI. For each covariate, we estimated the difference in mean baseline RAQ and reported the corresponding Wald 95% confidence interval and *p*-value.

Next, we evaluated whether the odds of baseline RAQ exceeding 28 was associated with pandemic period, by constructing a logistic regression model, adjusting for the same potential confounders. Estimated odds ratios and corresponding 95% Wald-based confidence intervals and *p*-values were computed for each covariate.

We assessed longitudinal changes in RAQ scores across pandemic time periods via generalized estimating equations (GEE). Analogous to the cross-sectional analysis, one model treated RAQ score as a continuous outcome, while the other used RAQ > 28 as a binary outcome. Since each participant completes the RAQ annually, we used GEE to account for within-individual correlation in repeated measurements over time. We employed an exchangeable working correlation structure, which assumes a constant correlation between an individual’s responses. Models adjusted for the same *a priori* identified potential confounders as the cross-sectional models.

The extent of missing data in this study was relatively limited. During preprocessing, we addressed missing values as follows: time-invariant covariates (race and ethnicity) were filled with any available renewal data (*n* = 136), and missing ADI values were imputed with each participant’s most recent non-missing value (*n* = 417). After preprocessing, all analyses were conducted using complete-case data. In the cross-sectional dataset, 1,161 participants were excluded, yielding a final sample size of 3,937; most missingness was in ADI (*n* = 515), chronic health conditions (*n* = 485), and race/ethnicity (*n* = 166). In the longitudinal dataset, 672 observations were excluded, with missingness primarily in chronic health conditions (308 of 7,187 observations) and ADI (197 of 7,187 observations). Given the relatively modest extent of missing data, we do not expect it to have affected the validity of our results.

Residual diagnostics were conducted to evaluate the influence of individual observations. A small number of influential cases were identified with RAQ scores of 7 (the minimum). Although these values were anomalous relative to the rest of the cohort, they were retained in all analyses because we could not rule out the possibility that they represented valid scores.

To assess the robustness of our findings, we performed two sets of sensitivity analyses. First, to evaluate sensitivity to the dichotomization threshold of the primary outcome, we re-estimated both cross-sectional logistic regression and longitudinal GEE models using alternative RAQ thresholds of >27 and >29. Second, to evaluate potential temporal confounding, we conducted sensitivity analyses using restricted comparison windows (P1 and P3) surrounding the 9-month P2 period (March 13, 2020 to December 11, 2020). For cross-sectional models, P1 and P3 were defined as the immediately preceding and following 9-month intervals, respectively (P1: June 13, 2019 to March 12, 2020; P3: December 12, 2020 to September 11, 2021), establishing symmetric time windows around the 9-month P2 period. For longitudinal GEE models, P1 and P3 were defined as 14-month periods (P1: January 13, 2019 to March 12, 2020; P3: December 12, 2020 to February 11, 2022) to appropriately accommodate the registry’s annual renewal cycle while still evaluating a restricted observation window.

## Results

Table 1 presents baseline demographic characteristics stratified by COVID-19 period. Our sample was predominantly female (65.7%), non-Hispanic White (70.2%), and over the age of 50 (66.7%). Most participants were without chronic health conditions (58.2%) and lived in low-disadvantage neighborhoods (ADI levels 1 or 2, 59.1%). Educational attainment was high, with 97.8% having some higher education. The groups were largely similar, though those enrolled in P1 were more likely to have been recruited through an email campaign.

**Table 1:** Demographic and clinical characteristics of C2C enrollees across enrollment periods.

Covariate	P1 (*N* = 3,751)	P2 (*N* = 121)	P3 (*N* = 1,226)
Sex			
Female *n* (%)	2,416 (64.4)	91 (75.2)	842 (68.7)
Male *n* (%)	1,335 (35.6)	30 (24.8)	383 (31.2)
Missing *n* (%)	0 (0.00)	0 (0.00)	1 (0.10)
Age (yrs) mean (SD)	57.1 (16.4)	55.8 (19.2)	54.9 (19.5)
Missing *n* (%)	42 (1.10)	0 (0.00)	45 (3.70)
Recruitment source			
Email *n* (%)	1978 (52.7)	17 (14.0)	150 (12.2)
Media/Online Search *n* (%)	477 (12.7)	38 (31.4)	416 (33.9)
Personal Network *n* (%)	583 (15.5)	27 (22.3)	248 (20.2)
Personal Physician *n* (%)	151 (4.00)	14 (11.6)	40 (3.30)
Other* *n* (%)	520 (13.9)	25 (20.7)	372 (30.3)
Missing *n* (%)	42 (1.10)	0 (0.00)	0 (0.00)
Race and ethnicity			
Non-Hispanic White *n* (%)	2,731 (72.8)	77 (63.6)	771 (62.9)
Hispanic White *n* (%)	205 (5.50)	11 (9.10)	79 (6.40)
Non-Hispanic Asian *n* (%)	257 (6.90)	14 (11.6)	192 (15.7)
Non-Hispanic Black *n* (%)	44 (1.20)	3 (2.50)	36 (2.90)
Other** *n* (%)	351 (9.40)	16 (13.2)	145 (11.8)
Missing *n* (%)	163 (4.30)	0 (0.00)	3 (0.20)
Education (yrs) mean (SD)	16.3 (2.70)	16.2 (2.80)	16.3 (2.90)
<12 yrs. *n* (%)	28 (0.70)	1 (0.80)	16 (1.30)
12–16 yrs. *n* (%)	2,265 (60.4)	63 (52.1)	726 (59.2)
>16 yrs. *n* (%)	1,396 (37.2)	54 (44.6)	483 (39.4)
Missing *n* (%)	62 (1.70)	3 (2.50)	1 (0.10)
Chronic health condition			
Yes *n* (%)	1,290 (34.4)	31 (25.6)	326 (26.6)
No *n* (%)	1981 (52.8)	89 (73.6)	896 (73.1)
Missing *n* (%)	480 (12.8)	1 (0.80)	4 (0.30)
Area deprivation index			
1 *n* (%)	1,111 (29.6)	42 (34.7)	394 (32.1)
2 *n* (%)	1,079 (28.8)	40 (33.1)	348 (28.4)
3 *n* (%)	666 (17.8)	24 (19.8)	211 (17.2)
4 *n* (%)	286 (7.60)	2 (1.70)	124 (10.1)
5 *n* (%)	165 (4.40)	3 (2.50)	88 (7.20)
Missing *n* (%)	444 (11.8)	10 (8.30)	61 (5.00)

This table displays summary statistics of 5,098 participants at time of enrollment, reporting mean (standard deviation) for continuous variables and count (%) for discrete variables. *Recruitment source defined as “Other” included postcard, multiple sources, none of the above. **Race and Ethnicity defined as “Other” included Hispanic Black, Hispanic Asian, NH/Hispanic American Indian or Alaska Native, Native Hawaiian or Other Pacific Islander, self-identified other, and multiracial.

Figure 1 illustrates the trajectory and distribution of baseline RAQ scores across COVID-19 periods. The top panel presents boxplots of baseline RAQ scores over 9-month intervals, highlighting a local peak during P2 (shown in pink). This peak represents the highest median relative to surrounding intervals, although the global maximum median RAQ score was observed at the beginning of the C2C registry. Notably, the P2 boxplot also shows the narrowest spread, with a higher 25th percentile compared to other intervals. The bottom panel displays the distribution of baseline RAQ scores within each period. While the distributions for P1 and P3 appear similar, P2 shows a notable shift toward higher RAQ scores. For example, 60.2% of participants enrolled in P2 had RAQ scores above 28, compared to 49.2% in P1 and 49.5% in P3.

Table 2 presents the results of the linear regression model estimating differences in mean RAQ at time of enrollment into the C2C. Tests for period differences in mean RAQ were not significant for any pairwise comparison. Specifically, we observed 0.70 higher mean RAQ in P2 relative to P1 (Est. 0.70; 95% CI: −0.08, 1.49; *p* = 0.079) and a smaller increase of 0.10 in P3 compared to P1 (Est. 0.10; 95% CI: −0.23, 0.43; *p* = 0.550), adjusting for covariates.

**Table 2:** Estimates of mean differences in RAQ scores at enrollment.

Covariate	Unadjusted		Adjusted	
	Est. Mean Diff (95% CI)	*p*-value	Est. Mean Diff (95% CI)	*p*-value
Period				
P1	Referent		Referent	
P2	0.87 (0.09, 1.65)	0.029	0.70 (−0.08, 1.49)	0.079
P3	0.31 (0.03, 0.59)	0.032	0.10 (−0.23, 0.43)	0.550
Age (per 10 yrs)	0.09 (0.02, 0.17)	0.019	0.00 (−0.10, 0.09)	0.926
Recruitment source				
Email	Referent		Referent	
Media/Online Search	0.66 (0.29, 1.04)	<0.001	0.76 (0.34, 1.18)	<0.001
Personal Network	0.77 (0.40, 1.14)	<0.001	0.68 (0.30, 1.06)	<0.001
Personal Physician	−0.51 (−1.33, 0.32)	0.232	−0.48 (−1.31, 0.35)	0.261
Other	0.45 (0.09, 0.81)	0.013	0.44 (0.05, 0.83)	0.028
Sex				
Female	Referent		Referent	
Male	−0.11 (−0.39, 0.17)	0.457	−0.17 (−0.45, 0.11)	0.236
Race and Ethnicity				
Non-Hispanic White	Referent		Referent	
Hispanic White	−0.34 (−0.98, 0.30)	0.302	−0.14 (−0.79, 0.50)	0.666
Non-Hispanic Asian	−0.38 (−0.85, 0.09)	0.109	−0.55 (−1.03, −0.06)	0.027
Non-Hispanic Black	0.09 (−0.82, 1.01)	0.839	0.16 (−0.76, 1.07)	0.735
Other	−0.60 (−1.07, −0.13)	0.013	−0.48 (−0.97, 0.01)	0.057
Education (per 5 yrs)	0.72 (0.49, 0.96)	<0.001	0.75 (0.51, 0.99)	<0.001
<12 yrs.	0.19 (−1.14, 1.53)	0.779	0.17 (−1.17, 1.51)	0.803
12–16 yrs.	Referent		Referent	
>16 yrs	0.77 (0.50, 1.04)	<0.001	0.76 (0.49, 1.03)	<0.001
Chronic health condition				
No	Referent		Referent	
Yes	0.34 (0.06, 0.62)	0.017	0.38 (0.07, 0.68)	0.015
Area deprivation index				
One	Referent		Referent	
Two	−0.01 (−0.34, 0.32)	0.949	0.12 (−0.21, 0.45)	0.463
Three	−0.14 (−0.51, 0.23)	0.465	0.12 (−0.26, 0.50)	0.526
Four	−0.36 (−0.91, 0.20)	0.206	−0.07 (−0.62, 0.48)	0.806
Five	−0.07 (−0.69, 0.56)	0.837	0.07 (−0.55, 0.70)	0.816

This table presents the results of a linear regression model estimating mean differences in RAQ scores at time of enrollment. Unadjusted and adjusted estimates are shown for each COVID-19 time period and adjustment covariates.

Following the evaluation of mean RAQ scores at enrollment, we estimated the odds of participants having a score exceeding 28 at the time of enrollment. Table 3 displays the results of the logistic regression model for the odds of initial RAQ > 28. Individuals enrolling during P2 were estimated to have a 60% increase in the odds of a high RAQ score compared to those enrolling during P1 [adj. Odds ratio (aOR) 1.60; 95% CI: 1.07, 2.40; *p* = 0.022], controlling for the same covariates as the previous model. This was followed by a return to baseline during P3 (aOR 0.97; 95% CI: 0.82, 1.14; *p* = 0.690).

**Table 3:** Estimated odds ratios for RAQ scores > 28 at enrollment.

Period	Unadjusted		Adjusted	
	Est. OR (95% CI)	*p*-value	Est. aOR (95% CI)	*p*-value
P1	Referent		Referent	
P2	1.70 (1.15, 2.51)	0.008	1.60 (1.07, 2.40)	0.022
P3	1.05 (0.91, 1.20)	0.515	0.97 (0.82, 1.14)	0.690

This table presents the results of a logistic regression model estimating the odds of reporting a RAQ score > 28 at time of enrollment. Unadjusted and adjusted estimates are shown for each COVID-19 time period.

Table 4 presents the estimates of within-subject changes in RAQ scores over time. During P2, mean within-individual RAQ scores increased compared to P1 (Est. 0.37; 95% CI: 0.08, 0.66; *p* = 0.011), whereas no significant difference was observed between P3 and P1 (Est. 0.12; 95% CI: −0.09, 0.33; *p* = 0.267), adjusting for covariates. Table 4 also provides the results of the GEE model examining the odds of reporting a high RAQ score (>28) within individuals over time. The within-individual odds of having a high RAQ score were 24% higher during P2 compared to P1 (aOR 1.24; 95% CI: 1.09, 1.39; *p* < 0.001) and 11% higher during P3 compared to P1 (aOR 1.11; 95% CI: 1.00, 1.22; *p* = 0.038), adjusting for the same covariates.

**Table 4:** Longitudinal GEE estimates of mean differences in RAQ and odds ratios for RAQ > 28.

Period	Unadjusted				Adjusted			
	Est. Mean Diff (95% CI)	*p*-value	Est. OR (95% CI)	*p*-value	Est. Mean Diff (95% CI)	*p*-value	Est. aOR (95% CI)	*p*-value
P1	Referent		Referent		Referent		Referent	
P2	0.37 (0.09, 0.65)	0.011	1.24 (1.09, 1.39)	<0.001	0.37 (0.08, 0.66)	0.011	1.24 (1.09, 1.39)	<0.001
P3	0.15 (−0.05, 0.35)	0.139	1.11 (1.01, 1.22)	0.032	0.12 (−0.09, 0.33)	0.267	1.11 (1.00, 1.22)	0.038

This table displays the results of two generalized estimating equation (GEE) models estimating mean differences in RAQ scores and odds ratios for RAQ > 28. Unadjusted and adjusted estimates are shown for each COVID-19 time period.

In sensitivity analyses evaluating alternative RAQ dichotomization thresholds (>27 and >29), we did not find evidence of qualitative differences in our results across either cross-sectional or longitudinal models. Similarly, sensitivity analyses conducted using restricted P1 and P3 periods were consistent with our primary findings.

## Discussion

The COVID-19 pandemic constituted a major public health crisis, resulting in substantial mortality and widespread societal disruption. It also provided an opportunity to examine how a global public health crisis may influence attitudes toward medical research. Its unprecedented scale meant that nearly all individuals in the United States were affected, through infection and due to precautionary lockdowns and other safety policies, creating a natural case study to investigate how perceptions of medical research changed in response. Access to longitudinal data from the UC Irvine C2C registry, which includes annual RAQ scores collected for years before the pandemic and continuing to the present, uniquely positioned us to analyze these changes.

To our knowledge, this is the first study to investigate longitudinal changes in research attitudes during a public health crisis. While previous studies have assessed attitudes toward clinical research during the pandemic using cross-sectional data (21–24), they did not examine within-subject changes over time. Our findings indicate that the period from March 13, 2020, when the U.S. declared a national emergency due to COVID-19, to December 11, 2020, when the first COVID-19 vaccine was approved in the U.S. (P2), was associated with higher RAQ scores both cross-sectionally and longitudinally compared to pre-pandemic scores (P1). These longitudinal changes are particularly noteworthy, as previous research has shown RAQ scores within individuals to be highly stable over time (10).

Contrary to our hypothesis, increases in RAQ scores post-December 11, 2020 (P3) were smaller than those observed in P2, with one estimate indicating no increase. We predicted that the rapidity in developing COVID-19 vaccines (25) and the profound positive societal effects the availability of those treatments had (26, 27) would result in sustained support and more positive attitudes toward research during this period. This may indicate not only that attitudes about research change, but also that they are highly susceptible to current events, personal experiences with public health policies, and media coverage of specific research programs. Similar shifts have been documented in response to high-profile regulatory events, such as changes in willingness to participate in research following controversial regulatory decisions (28).

While the reasons for the smaller increase in positive attitudes toward research during P3 compared to P2 are unknown and could be driven by unmeasured confounding factors, the broader climate of mistrust surrounding the COVID-19 vaccine may have contributed. Following its approval, debates over vaccine mandates, along with widespread misinformation about its efficacy and potential side effects, dominated public discourse (29). The environment of distrust in science, along with the diminishing urgency of the immediate COVID-19 crisis, may have contributed to the less pronounced increase in positive attitudes toward research observed during P3 relative to P2.

The fragility of increased positive research attitudes observed during this period underscores the need for sustained efforts from researchers to engage and educate the public to help build and maintain support for medical research. Tailored community-based initiatives and outreach events hosted by academic and medical institutions have been shown to increase trust in, understanding of, and willingness to participate in medical research (30–32). Furthermore, diversifying staff and leadership ensures that research centers reflect and resonate with the communities they serve and is an important strategy to increase clinical research participation among minority populations (33, 34). Finally, during an active public health crisis, transparent and accessible communication is essential to build and sustain the public trust (35).

Our study has important limitations. First, our sample includes only individuals who voluntarily enrolled in a research recruitment registry, introducing potential selection bias and limiting generalizability. In particular, our cross-sectional findings during P2 may reflect selection bias, as average monthly enrollment in the C2C registry fell to 13.4 participants in P2, compared with 83.4 in P1 and 21.9 in P3, likely due to the suspension of in-person recruitment. As a result, individuals who enrolled during P2 may have been those with particularly strong or favorable attitudes toward research, potentially inflating the average RAQ scores in this group. However, our longitudinal analyses, which track within-individual changes over time, provide evidence that the observed patterns are not solely due to compositional changes in the sample. Selection bias may also be evident in Figure 1, where initial RAQ scores at the inception of the C2C registry are notably high. This likely reflects that the earliest enrollees were primarily individuals already participating in studies and otherwise connected to the registry and its operators. In the first half of 2019, the C2C experienced a surge in enrollment due to a large-scale electronic health record-based email recruitment campaign (reflected in the enrollment counts for 9/13/18–6/12/19 in Figure 1). Direct email outreach to community members and patient populations is a routine, ongoing recruitment mechanism for the registry. While this specific institutional campaign substantially increased the volume of enrollees over a condensed timeframe, the enrollment protocol, inclusion criteria, and baseline survey assessments (including the RAQ) remained identical across all periods. Regarding generalizability, our results may not extend to non-registry enrollees, as our sample differs from the broader Orange County population from which it was drawn. Specifically, our sample overrepresents individuals from the least disadvantaged neighborhoods (ADI quintile 1) relative to the overall county (36). Nonetheless, registry participants are highly relevant to the research question posed in this study, as their attitudes may directly influence clinical research participation. Our sample is also largely limited to Orange County, CA residents; findings may not generalize to other areas of the country or the world. Orange County is wealthier and more educated than the rest of the US; it is also highly diverse, though the racial and ethnic makeup of the region differs from the U.S. overall (37, 38). The group enrolled during P2 was smaller than the other groups, due to its shorter duration and to the reduced ability to recruit to the registry through in-person outreach efforts, limiting precision. We cannot rule out unaccounted temporal confounding, as P1 and P3 spanned several years, during which other external events could have influenced RAQ scores. The COVID-19 pandemic, however, was the most significant health crisis across the studied time periods. Our time periods may not perfectly capture the effects we aimed to measure, as public awareness of the pandemic and access to the vaccine did not change instantly on the incorporated dates. Nonetheless, our time periods were chosen *a priori* based on significant dates and provide a meaningful framework for examining changes in research attitudes. Lastly, while the RAQ is validated and has a growing literature to suggest meaningful associations with research behaviors, we lack data on drivers of research attitudes, such as individuals’ perception of science, their sources of news or health information, or their personal experiences during the COVID-19 pandemic.

## Conclusion

We observed more positive attitudes toward biomedical research between the declaration of the U.S. national emergency due to COVID-19 and the first vaccine approval (P2), followed by a shift towards pre-pandemic levels post vaccine approval (P3). This pattern may suggest that increased awareness of urgent medical needs can enhance views of medical research, but also that these more favorable attitudes may be tempered by the easing of the immediate crisis and/or challenges related to distrust. These observations underscore the need for sustained efforts to engage and educate the public about medical research beyond moments of crisis, ensuring that awareness and support remain strong even in the absence of a global emergency.

## Data Availability

The data analyzed in this study is subject to the following licenses/restrictions: to protect participant privacy and comply with Institutional Review Board regulations, the raw datasets from the UC Irvine Consent-to-Contact (C2C) registry are restricted and not publicly available. The de-identified data used and/or analyzed during the current study as well as statistical/analytical code are available from the corresponding author on reasonable request. Requests to access these datasets should be directed to Sarah Schlund, sschlund@uci.edu.
